# Collectanea Medica: Consisting of Anecdotes, Facts, Extracts, Illustrations, &c.

**Published:** 1821-04

**Authors:** 


### COLLECTANEA MEDICA:

CONSISTING OF

### ANECDOTES, FACTS, EXTRACTS, ILLUSTRATIONS, &c.

*Relating to the History or the Art of Medicine, and the Auxiliary Sciences.*

Floriferis ut apes in saltibus omnia libant,
Omnia nos itidem depascimur aurea dicta.

### *Extracts of the Report from the Select Committee of the House of Commons on the Doctrine of the Contagion of the Plague, in* 1819.

*Jovis,* 25° *die Martij,* 1819.

Sir John Jackson, Baronet, in the Chair.

Sir Arthur Brooke Faulkner, called in; and Examined.

STATE the opportunities you have had of considering the plague, and the nature of contagious distempers in general, in the course of your practice?—The only opportunity I have had of seeing the plague was in the island of Malta.

On what occasion?—In the year 1813.

When the plague prevailed?—When the plague prevailed in the island.

What situation at the time did you hold?—I was physician to the forces. I was the only staff physician employed during the greater part of that service.

Was you there at the time it broke out first?—I was.

And attended as a medical man during the whole course of it?—Not during the whole course. I did not attend officially until the army became infected. I was not permitted when the natives only were infected.

But you was present?—I was present during the whole period of the plague.

And practised as one of the principal men of the army?—After the army became infected, I practised as physician to the forces.

What is your opinion respecting the mode in which the plague, in general, is generated and communicated?—I believe it is generated or produced by a contagion *sui generis*, quite peculiar and specific, and that it is communicated only by contact or close association with the person or thing infected.

What are your reasons for believing that it is communicated only by contact or association, and not by a certain state of the air?—My reasons are drawn from the course the plague took from its first entrance into the island of Malta, until its cessation. It was communicated in the first instance in the direct line of contact. It could be traced to have been propagated in the direct line of contact in the city of Valetta, and from the city into most of the *cassals* or villages, where any history could be obtained of its introduction.

Will you have the goodness to state the instances of its communication by contact, during your own experience at Malta, from your own knowledge?—The first case of the communication of the plague was, in my opinion, from a vessel, the *San Nicola,* in the harbour, to the family of a person of the name of Salvator Borg.

The vessel lay in what harbour?—It was lying in the harbour contiguous to the city of Valetta; the harbour is called Marsamuchetts.

State the circumstances?—To prove that it was from this vessel the infection was received, I shall crave permission to read a letter addressed by myself to the governor. I am not quite sure of the verbal accuracy of the letter. After the vessel arrived in the harbour in March, the whole town became extremely alarmed, and among the rest myself. I understood, (but this I cannot pretend to vouch for,) that several merchants had remonstrated against the vessel remaining in the harbour. Participating in the common alarm, I thought it my duty, though not called upon in my official situation, to represent the consequences that appeared inevitable in permitting the ship to lie there; and therefore, though not solicited, I communicated the following letter to the governor:

"*10th April,* 1813.

"Sir,—Although in offering the following observations relative to the means of preserving this garrison from the calamitous distemper with which it is threatened, I should not be altogether able to elude a charge of intrusion upon the province of the health-officer, yet, being the only physician to his Majesty's forces on this station, I trust your Excellency may be pleased to see that the contribution of my opinion at such a juncture (though it has not been called for), is not unjustifiably at variance with my duty. It was my intention to have had the honour of addressing your Excellency on this subject some time ago, and previous to the malady being reported to be so close in our vicinity. The melancholy fate of the surrounding countries which have lately fallen a prey to its ravages, left in my mind no doubt as to the expediency of such a proceeding. My purpose was, however, for the present over-ruled by certain of my friends, who could not be brought to believe that the evil was in the least likely to approach so near to ourselves. If credit can be placed in the report of the day, these expectations have proved illusory, and therefore I can no longer apprehend that my addressing your Excellency on this subject should be deemed improper. I shall consequently hasten to submit for your consideration a proposal, from which, obvious as it certainly is, I am led to think considerable advantage might still be derived for the protection of Malta against the introduction of plague. With this view I would suggest, that neither the harbour of Marsamuchetts, nor the island there usually allotted to quarantine, should, upon the present emergency, be accessible to any arrivals from suspected ports; and therefore that some more distant, yet commodious, place be sought out, which, after being insulated from all intercourse with the population, should exclusively be destined for the reception of all persons with any suspicion of the disease. Some information which I have lately received will not allow me to doubt, sir, that such a place may be found; and that the vessels, cargoes and crews of infected ships, could be disposed of in such a secure manner as to allow of the least possible risk. Unless called upon, I will not trouble your Excellency with any detail of particulars by which my proposal might be carried into effect. Whether the apprehensions about the existence of plague in our vicinity be sufficiently founded or otherwise, I cannot resist conviction that the measure I have been proposing would, in time of such peril, have been at the first of much importance. Assuming as a fact that there exists no longer any reason to doubt that the disease has made its appearance in the neighbouring quarantine ground, the dangers arising from the extreme proximity of that situation to so many adjacent shores, and to so very populous a neighbourhood as this, are, in my humble judgment, too obvious to require comment; since the points of invasion by so formidable an enemy are thereby so much more multiplied. How awfully has the experience of other times informed us, that in this, and in similar situations of exposure, the plague has found its way, in defiance of the most vigilant regulations. It is this reflection which has chiefly prevailed with me to submit the above proposition to your Excellency; by the prompt adoption of which, it appears to me, the best chance would be afforded of checking the approaches of a calamity, which even a little delay might baffle the best efforts to oppose. I hope, sir, the urgency of the occasion will atone for any imperfections in this letter attendant upon haste and anxiety; and that you will believe me, with the most zealous concern for the interests of his Majesty's army and the people here committed to your governance, your Excellency's most obedient, &c.

" A. Brooke Faulkner,

" Physician to the Forces."

Such was the letter I had the honour of addressing to the commander in chief.

Go on with your detail?—My apprehension of the disease finding its way into Valetta, from the lazaretto and plague-ship, arose from my knowledge that the persons appointed as guardianas were taken from the lowest part of the community, and paid, as it appeared to me, very inadequately, somewhere from 1s. 6d. to 2s. a-day; and having recollected, from reading the history of a plague that visited the same island in 1675, that it crept on shore from an infected vessel unobserved, I thought I was justified in entertaining the same fears on the present occasion. Accordingly, on the 16th of this month, six days after this letter was presented to his Excellency Lieut.-General Oaks, the first infected case occurred in the town of Valetta; it was the case of the daughter of Salvator Borg. She died with well-marked symptoms of the disease, I think on the 19th April. Two other persons of the same family died on the 2d May, all with well-marked symptoms of the disease.

Will you trace the communication between them and the vessel?—From the family of Borg it made its way in a direct line into the family of one Maria Agius, a school-mistress, who, together with others she immediately communicated with, were attacked by the plague; and all of whom (with some of her scholars, I heard,) were seized or perished with well-marked symptoms of the disease. I ought to revert to that part of your enquiry which is of most consequence, namely, the communication between the vessel and the town of Valetta. I hold it as hardly requiring proof that the disease should have found its way from an infected ship in the harbour, when I consider the apparent connexion between the cause and effect, arising out of the arrival of the vessel and the almost immediate verification of my prediction to the governor; and recollect, besides, that the island had not been infected for 136 years before. I consider these circumstances as conclusive. But, in the next place, some new linen was discovered in the house of Salvator Borg, which was confidently rumoured to have been brought from the infected vessel; and it was further stated, (but of this I have no certain authority,) that when the vessel returned to Alexandria, the infected place from whence it came, there were some bales missing.

Have you any reason to believe that the family infected, among whom the disease broke out, had direct communication with the ship; and what means have you of knowing it?—When I consider what appeared to me the imperfect state of the quarantine system at Malta, I can only say, I think it an event not improbable that some of the family might have got goods from this vessel.

Can you state that any communication took place, and what, between the family of Borg and the family of Maria Agius, where the disorder next appeared?—The families of Maria Agius and Borg were intimately acquainted with each other, and she was constantly employed in relieving the afflictions of the latter when taken ill of the plague.

From the family of Agius could you trace the progress of contagion to any other family?—For my own part I dropped the inquiry there: the *foci* of contagion became so rapidly multiplied, that it appeared to me impossible to carry the investigation in a direct line further in that populous city; but I am in possession of documents furnished to me by one of the captains of the lazaretto himself, a man of strict integrity, and many years employed in that official situation, showing that the contagion made its way in a direct line from Valetta into most of the infected cassals or villages: these documents I can produce, if required. In the next place, in pursuing the course of the disease by contagion, I should beg leave to remark, as a very important circumstance, that the means of its communication to the small contiguous island of Gozo, at a late period of the calamity, can be distinctly made out. A man belonging to an infected family in one of the cassals made his escape with a box of clothes into a neighbouring cottage; it was speedily found out that he had escaped, and he was accordingly apprehended and sent to the lazaretto. On his enlargement from the lazaretto, he returned to his cottage where he took this box of clothes, that had never been suspected to be there, but had been concealed; and he hired a boat and carried this box of clothes to the island of Gozo. The first family infected on the island was the family at whose house he arrived, and to which place he carried the box of clothes. It was a marriage present, I understood; and a priest acquainted in the family was one of the first victims; he died with well-marked symptoms of the plague. I have not had it in my power to trace the direct communication further.

Did the individual who conveyed the clothes take the plague; was he himself infected?—I am not prepared to answer that question, but I rather think he was. Many persons were not infected by the disease, that were taken from the very bosom of those families who all died of it.

Do you happen to know whether this person had communication personally with the individual who took the complaint, as well as by giving the goods?—He lived in the family; there was a marriage about to take place, or had taken place in it.

How long after he got this box of clothes was it before the disease broke out?—Within a short time.

What length of time were the clothes locked up in the box, between the time of their being infected and the box being opened, and the clothes given to the people in Gozo?—During the term of his quarantine, I believe somewhere about twenty days; I am not quite prepared to answer when it was opened. All I can say is, that the box was carried by this man to a cottage, and was concealed from those who went to take him to the lazaretto.

Do you know whether any person in the cottage where the box was deposited, took the plague?—I cannot answer that question.

Do you know whether there was any communication, any thing that could have conveyed the disorder from the ship *San Nicola* to the family, except the linen?—I am not aware of any thing; I rest my whole evidence on what I before specifically stated, namely, the tendency of the disease to propagate in a direct line, and the circumstance of my prediction being followed in four or five days by its consummation; and the collateral consideration of the island not having been infected with the plague for 130 years.

And you conceive that to have been a sufficient cause to account for the propagation of the plague?—I should think so. I beg to be understood in giving this evidence, that I was not present myself, and therefore I cannot speak with confidence as to the linen being found in Borg's house; I did not see the linen myself. As to the other circumstance of the disease propagating in a direct line to the cassals, I had the documents from one of the captains of the lazaretto.

You have stated that, in the family of Borg, and in that of Maria Agius the school-mistress, and also in the family in the island of Gozo, the different individuals died with well-marked symptoms of the plague: do you state the well-marked symptoms of the plague to have appeared, from your own individual knowledge, or from information derived from others?—From information derived from others. With respect to the first case, I had official information communicated by the head of my department; I received an official letter from him on the subject. With respect to Maria Agius, it was a fact so notoriously known, that I apprehend any evidence on this point would be unnecessary. With respect to the infection of the cassals in a direct line, my evidence, as I have stated above, rests on the written statement of one of the captains of the lazaretto, employed in the service during the plague of Malta.

Is he a medical man?—It is not necessary he should be so to fill that office.

With respect to Gozo?—With respect to Gozo, I have the information of some respectable authority, but I do not recollect his name: it is, however, a well-known fact.

Then, in point of fact, not one of these cases in which you state the symptoms to have existed, was from your own personal observation?—Not one. I was not officially employed; I offered my services in the native hospitals; but they were not received, on this ground, that they might be wanted for the army.

Had you any intermittent or other fever at Malta, during the plague?—I had none under my own immediate eye or care; but I understood there were a few, and those very mild, principally occurring, or indeed altogether occurring, towards the autumn or the latter end of the year 1813; but, as I have not seen any of the cases, I can only speak from hearsay.

Did those instances occur before the plague broke out?—They had, I understood, been occasionally numerous in preceding years.

The instances you state to have prevailed before the plague broke out, were they the marsh fevers?—I am not aware that any had occurred the year before; i. e. I cannot specify myself, on my own authority, that any occurred the year before; but I understood that they were of frequent occurrence in every year.

Was the plague particularly prevalent in the places where the marsh fevers prevailed?—Not at all particularly prevalent where the marsh fevers were most generally produced.

Is there any similarity between the marsh-fever in Malta and the plague?—I never could trace any series of symptoms that could lead me, in the slightest degree, to suspect any identity between them; but I have known the plague to personate, in certain symptoms, almost every possible form of fever, and I have known it to be entirely free from every kind of fever: there is no certain type to which it can be affixed.

Do you mean to say that there is no distinct symptom that marks the plague as distinct from every other fever?—I do mean to say, that fever is not an essential attribute of the plague; it is frequently mortal where there is no fever. It is an extraordinarily anomalous disease, which defies (I should rather say, has almost defied,) definition. Dr. Cullen, I conceive, has defined it best; but even his definition is not a correct one. In fact, I may say, it has hitherto baffled nosologists scientifically to define it.

Are there not symptoms that distinguish the plague in a manner that cannot be mistaken?—There are symptoms which are sometimes called characteristic symptoms, but they are not constantly present. The most frequent and constant symptom was a peculiar cast of the eye, and a certain appearance of the tongue; the eye had the appearance described by Russel, of a muddy dull colour; I believe that to be the most frequent and most characteristic symptom of the plague. As to buboes, carbuncles, and appearances on the skin, they are not constant. In many of the most fatal cases, the patient perished before the buboes or any other eruption made their appearance; many without any other appearance than that of the eye.

What was the state of the pulse?—The pulse had been felt occasionally, through a tobacco-leaf, and was in many instances extremely rapid. The fine for feeling a pulse was several days' quarantine; if any medical man had felt a pulse, he was subject to this quarantine.

Can you show that prompt separation has been effectual in securing persons from contagion?—I can, in many instances: the instances would be difficult to detail, they are so numerous.

Did the disease extend to Sicily, or the neighbouring islands?—It did not extend to Sicily, in consequence of the prompt precautions that were adopted: and, had there been the same at Malta, my persuasion is that the disease would have been resisted *in limine*.

Were quarantine restrictions found effectual in resisting the plague at Malta?—Wherever proper quarantine restrictions were imposed with firmness, steadiness, and promptness, they seemed to be altogether effectual in preventing the extension of contagion; but the quarantine system seemed to me so extremely lax from the beginning, for several months, that it would have been next to impossible the disease should not have been widely disseminated through the island. I may enumerate as instances of this laxity in our quarantine system at Malta, that there was no complete census taken of the population, with a view of detecting cases of infection, until, I think, the 19th May, 1813. There was not a complete and sufficient corps of trusty guards until the month of August; the people were not shut up in their houses until, I think, the month of August: that is, not universally shut up; probably partially. It is notorious that contact constantly took place in the street, previous to the organization of this corps of guards, and the shutting-up of the inhabitants in their houses. I have official documents to prove all these points I have been just stating.

Was the plague ever known to be received in the lazarettos?—It has been known. I have heard many instances related to me by the Maltese, and here is evidence of one: I have brought the title-page of a book, which represents a monument raised to the memory of a grand master for having arrested the disease.

In what year?—1743.

Do you know any thing of the introduction of the former plague that ravaged Malta, and when did it occur?—I beg to refer to a paper I published on the disease, during my engagement on the plague at Malta, which was communicated to the Edinburgh Medical and Surgical Journal, and published 1st April, 1814. The passage I wish to read to the Committee, is this: "It is somewhat remarkable, that the history of the introduction of the plague, when it made such great ravages on the last occasion on the island, about a century ago, was nearly similar to what is circulated of the present; being attributed to some linen brought from a Levant vessel by a Maltese shopkeeper, which, after producing the disease in all those who first came in contact with it, ultimately disseminated the malady throughout the whole population."

During the late plague at Malta in 1813, was the disease arrested when the quarantine system was rigidly acted upon?—It was: from the moment that an adequate and regularly-organized police was established, and the inhabitants shut up in their houses, and other strict measures of quarantine enforced, (which was the case at a very late period,) in the month of August, the plague did rapidly decline.

How was the disease stopped at last?—By what I have just stated; by the organization of a sufficient corps of trusty guards and police restrictions, and by shutting the inhabitants up in their houses. I could name a number of other circumstances connected with the means of preventing it.

How were the medical attendants preserved?—With respect to the military hospital, of which I can speak from experience, the hospital in which I attended, (the pest-hospital,) they were, in my opinion, preserved by wearing a dress of oiled silk, which prevented the possibility of any contact of infected matter with the skin, and probably also by its promoting free and copious perspiration, and in consequence preventing absorption.

When was the plague stopped; at what time?—I am not quite sure whether it was in December.

Can you state when it began to decline?—It began before the month of August.

What do you conceive the hottest period of the year at Malta?—I am not prepared to answer that question; I believe the month of August. I have kept a thermometrical table; but, having it only for one year, the results of my observations are probably insufficient.

What was the temperature of the atmosphere at the period when you conceive the decline to have been apparent?—The 16th July appears to be the day when the plague was at its height; sixty-seven died that day; the thermometer was at 81 at four o'clock in the afternoon; in the morning at six o'clock at 77, and at ten at 81½. The next day, thirty-six died; the thermometer was at 82, and in one part of the day at 83. On the 18th of the month, fifty died; the thermometer was in the course of the day at 81. On the 29th, forty-one died; the thermometer was at 79. On the 20th of the month, forty-three died.

State the period at which there was a sensible decrease?—I think from the 16th of July there was an average decrease, though a very irregular kind of decrease; but the thermometer was rather higher than lower.

How far was the establishment of the police precautions to which you have alluded coincident with the decrease of the plague, with the visible decrease of the plague?—This question requires a cautious answer. The gentleman appointed at the head of the police begun to organize his system on the 3d July; the disease extended its ravages after he had organized his system in some degree; but it was not perfectly organized till the 2d August,—*i. e.* it did not shut the people up in their houses, nor was there an absolute prohibition of contact; but, as soon as it did enjoin absolute prohibition of contact, and shutting the people up in their houses, the disease declined.

Then it appears the decrease was not very visible till these precautions were put strictly in force?—Not so visible.

State, from the period at which they were strictly enforced, namely, the 2d August, what was the average decrease from that time?—On the 2d August, 50 persons died; on the 3d August, 48; on the 4th, 27; on the 5th, 47; on the 6th, 43; on the 7th, 35; on the 8th, 37; on the 9th, 24; on the 10th, 26; on the 11th, 28; on the 12th, 26; on the 13th, 31; on the 14th,—

Instead of putting the result of every day, look at your table, and see when the decrease became considerable?—I have brought it down since the 16th July until 14th August, to less than one-half; I have brought it down to 31 instead of 67, in less than a month.

From the 16th August did it go on gradually decreasing, till it disappeared?—It went on decreasing on the average, but not regularly.

At what period did it finally disappear?—On the 19th October: in my note of this day is stated the last occurrence of a case of plague in Valetta.

Did it continue in other parts?—In some of the cassals for a considerable time.

Have you any doubt whatever, that the decline of the complaint was produced by the prohibition of intercourse among the patients?—I feel satisfied that it was very much owing to the prompt measures of police; and my reason is, that the thermometer rose inconsiderably in point of fact, while the disease was decreasing fast.

Is there any other cause to which you can attribute the decrease and cessation of the plague?—I really do not see any other cause.

In your observation of the plague, has it appeared to you that it subsists only in a given temperature, neither in very great heat or very great cold?—During my residence at Malta, it did not appear to me that the temperature of the air had any thing to do with it. In my own opinion, I do believe that a very high or a very low temperature would check it.

Will you state the opinion you entertain, from the best sources derived, what degree of heat is not consistent with the plague?—I can only speak from my reading on that point. I believe that materially below 60, or probably at 60 degrees of heat, it cannot subsist; but it is bare conjecture.

You had never any opportunity of observing the plague except at Malta?—No.

Was there any thing remarkable in the state of the thermometer at the time the plague broke out at Malta?—Nothing, I believe.

What was the temperature at that time?—The thermometer, on the day the first case was reported to have taken place, was at 64; that was on the 16th April.

What was the state of health in the island before the plague broke out?—To the best of my knowledge, nothing remarkable. If there was any thing remarkable with regard to its climate, it was that there was nothing very remarkable, for the people were wondering that there was nothing remarkable in the state of the air to produce plague:

there was nothing anterior to the breaking out of the plague at all leading to any reason why it should exist, unless by contagion.

At what time did the first case of plague appear among the soldiers? —Without referring to my official letter, I cannot exactly state.

What month was it?—In June.

Was the soldier who was first attacked with plague, in barracks or quartered in the town?—I shall be under the necessity of consulting my medical register; but I have no doubt he was in barracks.

Could any communication, personal communication, be traced between the soldier first infected and any other soldiers who were afterwards infected?—I do not know that any had been attempted to be traced; for, when soldiers live in the gregarious manner they do, it would be in vain to make the inquiry.

Were the soldiers kept within their barracks previous to the appearance of the plague among them, and their communication with the inhabitants prevented?—In some of the barracks it was prevented, in some not; and it is material, in order to prove the contagious property of the disease, that, in those barracks where a strict quarantine system appeared to be kept up, the plague was excluded, though they were in an unhealthy part of the town; whereas, in other places that were more elevated and airy, but where there seemed not to be the same precautions observed, the disease was brought in.

Then the soldier first infected was in one of those barracks that had a more free communication with the town: had you the charge of the sick in the barrack in which the first case occurred?—I had partly the charge of them in this barrack, but not in the first case.

When did your charge of the plague patients commence?—It was relative to that point I wished to consult my register; but, as far as I can state with confidence, I was not employed officially to prescribe till De Rolle's regiment was infected. I think I was called in to see some other cases, but I had them not under my charge.

Were you yourself, in attending the sick, those who were ill of the plague, in contact with them?—Personally close to them, as nearly as it was necessary for me to approach them.

Had you the plague?—I believe not; though I have some doubt respecting that.

Who were the persons employed under you in the care of the sick?—Orderlies and such like.

Did any of them catch the plague?—Not one.

Were they necessarily in contact with the individuals who had the plague, and with their clothes and bedding?—Necessarily.

Were any precautions taken with the view of preventing them from catching the plague in the discharge of their duties?—Oiled-silk dresses were enjoined, by command, to be worn by every person in attendance about the sick.

Was it complied with?—It was, I believe, in point of fact. They were also enjoined a prompt ablution after touching the infected; they were obliged to wash their hands. In short, every means were adopted to prevent their catching the infection, with the addition of ablutions.

Was one of the precautions rubbing the body with oil?—It was; but there are the best reasons for supposing that it had no share in preventing the infection.

What reason?—It had been employed with all the attention possible in the garrison, but yet the disease made its way. It had been employed by those who attended in carrying out the dead, and who, I believe, almost all perished: there were very few instances of such persons who did not perish.

When you describe the plague as contagious, do you mean that it is communicated by the breath?—I am not prepared to say whether it may not, by closely inspiring the breath of an infected person; but this, in my judgment, is a kind of contact. My opinion is, that it is principally communicable by the touch, but I think it can be communicated in the former way also: few would be hardy enough to try the experiment.

If it can be communicated by the breath, how can wearing oil-skin dresses or ablution prevent the breath of the infected persons getting into the mouth or nostrils from the infected?—We know that, at a certain distance in other diseases, the contagion from fomites may be so diluted by the atmosphere as to become innoxious.

Was the progress of the plague among the troops rapid?—It was not rapid.

Not so rapid as among the inhabitants?—Not at all: in all, not above twenty, I believe, died up to the middle of October.

The progress of the disease, I mean?—In some it was rapid, and in some it was not; there was every variety.

What was the per centage of those in garrison attacked?—I am not prepared to say.

Was it a greater or less proportion than the inhabitants?—A less proportion.

The precautions applied to the troops were more rigid than those that could be applied to the population?—Certainly. I was going on to state some circumstances as to the degrees of precaution used in each of the military barracks. The Sicilian regiment, though situated in a very infected part of the island, a place called Florian, escaped by the promptness and vigilance of Colonel Rivarolla. De Rolle's regiment, which was in the healthiest spot, was invaded by the disease, and evidently, in my opinion, in consequence of their barrier admitting a contact with persons on the outside. It was a barrier at which you could shake hands with any body on the outside. In the 14th regiment, which was near the most unhealthy part of the town, there was but one person suspected, and his disease was immediately arrested. The public prison, and public general hospital, escaped. The convents in Valetta escaped, with the exception, I believe, of one; and the introduction of the disease to that one was accounted for. The prison and these public institutions escaped, I conceive, very much by the voluntary attention paid by their inhabitants to a strict system of quarantine.

Did the plague cease in the military hospitals before it ceased in the town?—I am not prepared to answer that question.

Were the soldiers in barracks prevented from holding communication with the town, after the plague had ceased in the barracks?—I believe so: that would greatly depend upon the commanding officer.

I ask you whether, in point of fact, they were?—Throughout the whole, they were interdicted as far as possible; the commanders of regiments issued orders to prohibit intercourse, but they were not strictly obeyed.

Did the plague find its way into any of the barracks or regiments where these orders were not strictly observed?—It got into De Rolle's regiment particularly.

You have stated that these three cases of Salvator Borg, Agius, and Gozo, were cases you received from information. Now, I observe, in many cases that came under your own knowledge, it did not communicate to persons. Do you not consider that the natural time of the decrease of the plague, is the fall of the year?—It is my own opinion that it would cease if the temperature was low: I think that the plague is incompatible with a certain temperature, high or low.

Then it is not the particular period of the year?—No.

You stated it to appear prior to August, but the restriction was not till the 2d of August?—Not in its full rigour, but an improved system was acted upon in July.

The ship Nicola returned to Alexandria with her whole cargo, did she not?—If I may except those bales which were rumoured to be missing.

She was not allowed to land her cargo?—No.

Who navigated her back?—Probably the remaining part of the crew.

Was she not navigated back by Maltese?—She might.

Do you know whether they took the plague?—They arrived in safety.

Did they who assisted in landing the cargo?—I believe so.

Are there any eruptions in the skin, in the plague?—If we can call eruptions, what are termed blains and carbuncles.

Do you consider a bubo or carbuncle to be an eruption?—I should think so; it is freely applicable to the term: its etymology is from *erumpo,* which is to break forth.

Does not eruption mean a cluster of pimples?—In common acceptation it may.

Do you think the plague comes under the description of Exanthemata?—I think it does. I believe that Dr. Cullen has given the best account of the disease.

You have stated that the contagion in plague is what is called *sui generis;* do you consider it different from contagion in the small-pox?—Different from every other known contagion.

Have you ever heard of the plague in England?—I have read of it.

In what year?—The last was in 1665.

Have you ever heard of plague since?—Never, as imported into England.

Do you consider the plague can be propagated from goods as well as persons?—I think so.

Have you any reason for thinking why the plague has not been introduced from goods in the quarantine establishments?—In the first place, quarantine restrictions since 1709 have been a great deal more rigid; indeed, they did not exist in England at all previous to that period. I conceive that the intensity of the contagion may have been greatly blunted by the length of the voyage, and the length of time that passes after the shipment of goods. Besides, we know that other countries have a good system of quarantine, which is in favour of the plague not being imported here.

Do you consider the plague of 1665 to be the true Levant plague?—From the description I have read of it, I am inclined to think so.

Do you consider the reason why the plague has not been introduced in England, has been more from the length of time in the voyage, than the quarantine establishment?—I mean both conjunctively,—viz. the time and the means used to free goods from contagion and to expurgate infection.

How do you account for the expurgators never having taken the plague?—I cannot account for that, but by collateral considerations.

What are those?—1st. That we have observed, in other countries, the disease has not taken place for a long series of years; not for 130 years in Malta. 2dly. We do not know what the circumstances are that constitute aptitude in the receiver, sufficiently, to know why the plague has not been received into the lazarettos since 1664. But, 3dly, it does not follow, because it has not been received into the lazarettos since 1665, that it may not, by some fortuitous concurrence of circumstances, occur again here.

Are you acquainted with the opinion of the ancients respecting the plague?—I am. It has been stated that the ancients were not acquainted with contagion, but I can adduce instances, from the medical writers and the poets, to the contrary; I can produce instances from both the Greek and Roman writers and poets.

Does Hippocrates mention it?—He does not. He had not the experience to determine the point; but, with respect to the authorities that do speak of contagion, I shall beg to refer to the following, viz.

Συνδιατρίβειν τοῖς λοιμώττουσιν ἐπισφαλὲς ἀπολαῦσαι γαρ κίνδυνος, ὥσπερ ψώρας τινος ἢ ὀφθαλμίας.—Galen, lib.i. ch. 2. *de different. Febrium.*

Διὰ τί ἀπὸ μὲν νόσων ἐνιῶν νοσοῦσιν οἱ πλησιάζοντες, ἀπὸ δὲ ὑγιείας οὐδεὶς ὑγιάζεται.—Aristotle, *Prob.* lect. vii. 1.

Δέος δε ξυμβιοῦν τε, καὶ ξυνδιαιτᾶσθαι, οὐ μεῖον ἢ λοιμῷ· ἀναπνοῆς γὰρ ἐς μετάδοσιν, ῥηΐδιη βαφή.—Aretæus *de Elephantiase.*

Infecti quasi valitudine et contactû.—*Annal.* Tacit. b. 6 and 7.

Postea curatio ipsa et contactus ægrorum, vulgabat morbos.—Liv. 25 and 26.

Contagion is clearly expressed in the last eight lines of the third *Georgic* of Virgil; likewise in the first *Bucolic*, verse 52; where these words occur:

Nec mala vicini pecoris contagia lædent.

There are numerous other authorities.

